# The British Columbia Nephrologists’ Access Study (BCNAS) – a prospective, health services interventional study to develop waiting time benchmarks and reduce wait times for out-patient nephrology consultations

**DOI:** 10.1186/1471-2369-14-182

**Published:** 2013-08-29

**Authors:** Michael E Schachter, Alexandra Romann, Ognjenka Djurdev, Adeera Levin, Monica Beaulieu

**Affiliations:** 1 British Columbia Provincial Renal Agency, 700, 1380 Burrard Street, Vancouver BC V6Z 2H3, Canada

**Keywords:** Health services, Administration, Wait times, Nephrology, Chronic kidney disease, Physician engagement, Change management, Waiting time benchmarks

## Abstract

**Background:**

Early referral and management of high-risk chronic kidney disease may prevent or delay the need for dialysis. Automatic eGFR reporting has increased demand for out-patient nephrology consultations and in some cases, prolonged queues. In Canada, a national task force suggested the development of waiting time targets, which has not been done for nephrology.

**Methods:**

We sought to describe waiting time for outpatient nephrology consultations in British Columbia (BC). Data collection occurred in 2 phases: 1) Baseline Description (Jan 18-28, 2010) and 2) Post Waiting Time Benchmark-Introduction (Jan 16-27, 2012). Waiting time was defined as the interval from receipt of referral letters to assessment. Using a modified Delphi process, Nephrologists and Family Physicians (FP) developed waiting time targets for commonly referred conditions through meetings and surveys. Rules were developed to weigh-in nephrologists’, FPs’, and patients’ perspectives in order to generate waiting time benchmarks. Targets consider comorbidities, eGFR, BP and albuminuria. Referred conditions were assigned a priority score between 1-4. BC nephrologists were encouraged to centrally triage referrals to see the first available nephrologist. Waiting time benchmarks were simultaneously introduced to guide patient scheduling. A post-intervention waiting time evaluation was then repeated.

**Results:**

In 2010 and 2012, 43/52 (83%) and 46/57 (81%) of BC nephrologists participated. Waiting time decreased from 98(IQR44,157) to 64(IQR21,120) days from 2010 to 2012 (p = <.001), despite no change in referral eGFR, demographics, nor number of office hrs/wk. Waiting time improved most for high priority patients.

**Conclusions:**

An integrated, Provincial initiative to measure wait times, develop waiting benchmarks, and engage physicians in active waiting time management associated with improved access to nephrologists in BC. Improvements in waiting time was most marked for the highest priority patients, which suggests that benchmarks had an influence on triaging behavior. Further research is needed to determine whether this effect is sustainable.

## Background

Delays in access to medical services are an important issue for publicly funded health systems. In Canada, 57% of patients waited more than four weeks for a specialist appointment in 2004, which is worst among Australia (46%), the United Kingdom (40%), Germany (23%) and New Zealand (22%) [1]. Such barriers within the primary to specialist care-continuum may prolong exposure to time-sensitive disease in the absence of expert management.

The Canadian government addressed the problem of delayed access to healthcare in 2004 through the $5.5 billion Wait Times Reduction Fund [2]. The program’s objective was to reduce delays for medical procedures, cancer treatment, and diagnostic imaging. The interval of waiting that it targeted is the period between booking procedures and their completion, which is known as *waiting time 2*[3,4]. The preceding period of delay, between referral and first assessment by a specialist, is known as *waiting time I*. Waiting time I is the greatest concern for family physicians, among whom 50% consider it unacceptably long [5]. The Canadian Medical Association and Health Canada began efforts to address waiting time I in 2010. A toolkit for streamlining referral processes was developed, and a resource to highlight current referral reform projects has been compiled (http://www.cma.ca/referrals).

Chronic kidney disease (CKD) is prevalent with approximately 2 million Canadians affected [6], and for patients with progressive CKD, timely access to nephrologists is vital. Early management of high risk CKD by a nephrologist has been shown to slow progression to End Stage Renal Disease (ESRD) [7-13]. Recent implementation of automatic estimated glomerular filtration rate (eGFR) reporting by laboratories in Canada and abroad has improved screening, but increased the volume of both appropriate and inappropriate referrals, which likely prolongs queues [14-16]. Current guidelines make suggestions regarding who should be referred to a nephrologist, but do not indicate appropriate timeframes for assessment [17].

In 2006, the Federal Advisor on Wait times recommended the development of targets for appropriate waiting [18]. This process has been undertaken for paediatric nephrology, though not for adult kidney disease, which is more prevalent [19]. The BC Nephrologists’ Access Study (BCNAS) was designed as a Provincial collaboration to study wait time I. We conceived of a change management strategy based on physician engagement and wait time benchmark development. We hypothesized that involving local physician stakeholders in generating consensus waiting time I benchmarks, would reduce the wait for outpatient nephrology consultations. To test the hypothesis, we: 1) Conducted an environmental scan to measure wait times in the Province; 2) Engaged nephrologists and referring physicians to develop maximally-recommended wait time targets; 3) Encouraged pooled triage (patients assigned to first-available nephrologist in group practice) where possible; and 4) Re-measured wait times post-introduction of targets.

## Methods

### Study design

The BCNAS was a prospective, pre-, post-intervention, interrupted time-points observational, design. Data collection was performed over two-cycles (January 18–28, 2010 and January 16–27, 2012) to determine the impact of the intervention on wait times. Physician, region, and patient anonymity were maintained. BCNAS was approved by the University of British Columbia Research Ethics Board.

### Setting and participants

We sought to collect data from the 12 nephrology practice groups in British Columbia (BC). Provincial nephrologists collectively service an estimated catchment population of 4.6 million people as of July 01, 2011 [20]. All nephrologists in BC are affiliated with one of the 12 groups, which are organized within the Provincial health system into 5 geographic Health Authorities (HA). The BC Provincial Renal Agency (BCPRA) oversees administration of renal services in the Province. Representative nephrologists and administrators from all 5 Health Authorities sit on the BCPRA Executive and Medical Advisory Committees (MAC), which meet regularly. Using this infrastructure it is possible to undertake Provincial Initiatives, which span health regions. Data for our study was thereby collated and analyzed centrally by BCPRA statisticians. Nephrologists who agreed to participate allowed their private office Medical Office Assistants (MOAs) to perform data-entry. MOA participation was recognized with an honorarium.

### Data collection

**Figure 1:**
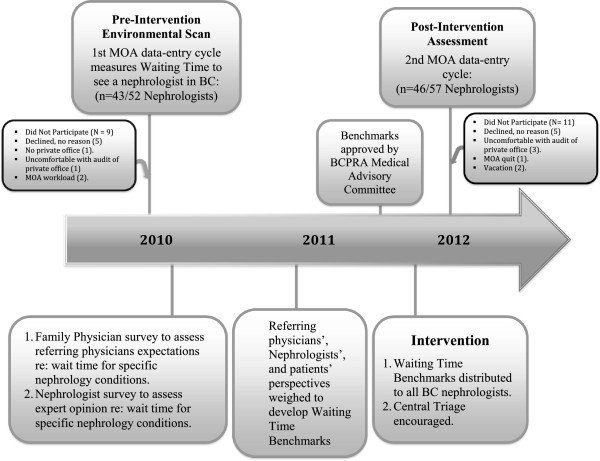
Study timeline.

**Figure 2:**
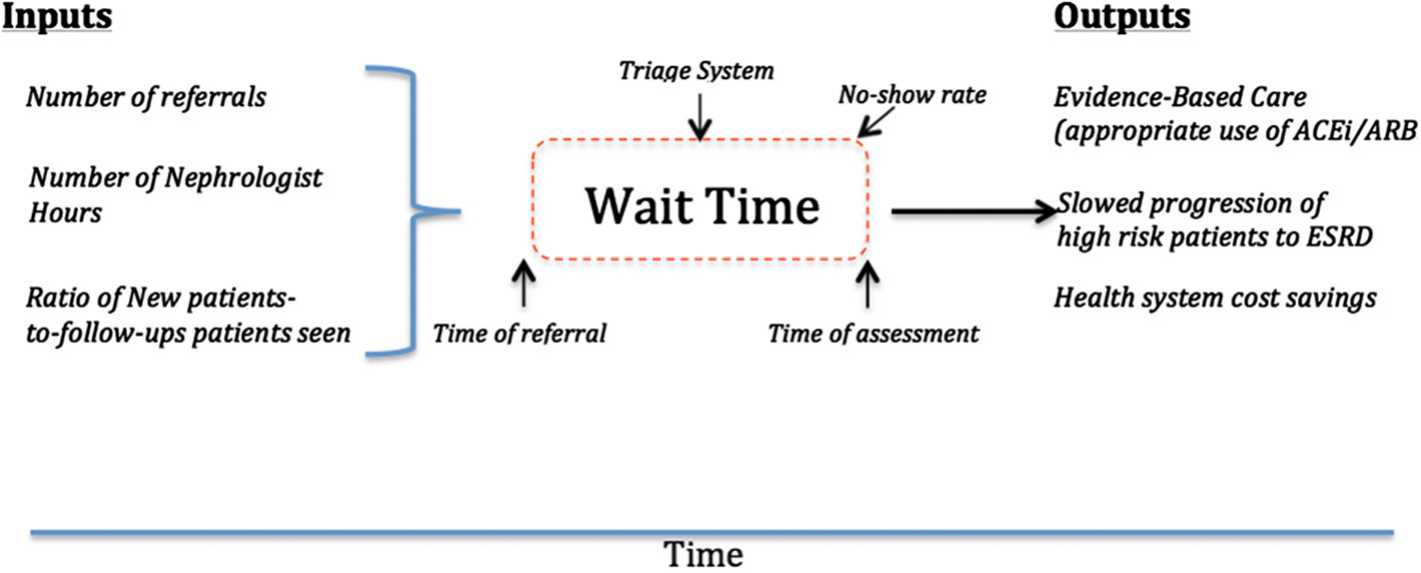
Schematic representation of multiple factors that may impact upon provincial out-patient nephrology waiting time.

Standardized data collection forms were used to abstract information about nephrologists’ practices as well as demographic and clinical data pertaining to new patients. Specifically, we recorded patients’ age and sex, renal function, proteinuria, reason for referral, and how many times they were re-booked before being seen. Nephrologist participation and study structure are outlined in Figure 1. To allow inferences about planned and actual wait times, a distinction was made between 2 types of new patients that were identified during the 2-week study window. *New patients* were defined as those not previously known to the nephrologist, or those last seen more than 24 months ago. Wait times were defined as the interval between fax receipt of the referral letter and assessment of the patient. Factors presumed to impact upon Provincial wait times are shown in Figure 2.

Reasons for referral were indicated by MOAs on the data-collection form. This information was confirmed through manual audit by a nephrologist (MS) and used to categorize referrals into 4 priority categories. Dialysis patients referred from another nephrologist for transfer of care were considered new, because they required a full consultation, but were not placed into a priority category because dialysis therapy is presumed to be ongoing and therefore less time-sensitive.

### Intervention

The intervention consisted of a physician-led Provincial initiative with goals to measure wait times, develop maximally recommended wait time targets, and promote targeted reductions if needed. The wait time issue was broached using an initial presentation and preliminary survey at the BC Nephrology Days Conference, Nephrologists’ consensus meeting in November 2009.

Through further face-to-face meetings and surveys, we used an iterative process modeled on the modified Delphi technique. Input from stakeholders including BC nephrologists and Family Physicians (FP) was used together to develop condition-specific wait time recommendations. This process also stimulated an ongoing Provincial dialogue on wait times, which included advancing use of pooled triage and telephone advice for non-progressive kidney disease.

Following the pre-intervention, environmental scan (first MOA data-collection cycle Jan 18–28, 2010) the most common reasons for referral were identified and used to develop two physician surveys: one for nephrologists and the second for FPs. The nephrologist survey was administered at the 2010 BC Nephrology Days meeting, which the majority of provincial Nephrologists attend. Non-attendees were asked to participate online. Nephrologists were asked to suggest appropriate wait times for common referral categories, 1st from the physician’s medical perspective, and then to assume the perspective of a patient. This was done to weigh-in the patient’s unmeasured burden of waiting when they have been told they need to see a kidney specialist.

The other survey was given to FPs attending a one-day course in nephrology. It was identical to the survey given to nephrologists, except did not ask FPs to assume the patient’s perspective. This was done to weigh-in the need for timely management advice among referral-base colleagues.

The following rules were developed by the study investigators (MS, MB and AL) to be used to develop waiting time I benchmarks:

1. Use the median nephrologists’ medically recommended wait time except when rules 2 or 3 apply.

2. If >1/3 of nephrologists revised wait times downward when asked to assume the patient’s perspective, reduce benchmark one wait time category sooner.

3. If 50% of GPs believe the wait should be shorter than determined by rules 1 and 2, revise 1 wait time category sooner.

With this strategy, 4 priority categories were developed for out-patient nephrology cases. Priority I includes referrals that should be seen within 3 weeks; priority 2 within 6 weeks; priority 3 within 12 weeks; and, priority 4 within 24 weeks.

Once the prioritization process was completed, to confirm consensus, the final step in our iterative benchmarking method included approval of benchmarks by BC Nephrologists at the BCPRA Medical Advisory Committee meeting (June 3, 2011). These benchmarks were then distributed to all BC Nephrologists. In December 2011 all BC nephrologists were contacted to remind them of the maximally recommended waiting time benchmarks. The majority of physicians had participated in the benchmarking exercise. Hard-copies of the recommendations were provided with the study package for the (post-intervention) 2nd MOA survey to serve as a reference sheet to be used during triage of new referrals.

### Outcome measures

The primary outcome was the proportion of new patients that were seen within recommended wait time targets. Additional metrics included the change in wait times following introduction of Provincial wait time targets, and the change in planned wait time (at the time the referral request is received). Descriptive statistics pertaining to Provincial nephrology referrals and an analysis of regional variability in wait times and referral acuity were also pre-determined.

### Statistical analysis

The underlying distributions of continuous variables were assessed using the Shapiro-Wilk test for normality. None of the continuous variables were normally distributed; hence, they are presented as median with interquartile range. Comparisons were made via the Wilcoxon χ2-test. Statistical software used was SAS, version 9.1 (SAS Institute, Cary, NC, USA).

## Results

### Nephrologists and family physicians

Nephrologist participation included 43 of 52 (83%) in 2010 and 46 of 57 (81%) in 2012 (Figure 1). Ten of 12 (83%) nephrology practice groups had at least one member participate in 2010 and 2012 MOA data-collection cycles. Among 2012 nephrologist respondents, 83% had also participated in 2010. Table 1 shows characteristics of participating nephrologists as well as referred patients. Thirty-five of 43 FPs (81%) attending a one-day course in Nephrology participated in the FP survey.

**Table 1:** Characteristics of nephrologists and patients referred in 2010 and 2012

**Group, characteristic**	**2010**	**2012**	**p value**
**Nephrologists**	n = 43	n = 46	--
Age, yr (%)^*^			
<40	-	37	
41-50	-	33	--
51-60	-	13	
>60	-	17	
Practice size (%)			
<300	10	37	
301-500	16	35	P < 0.001
>500	75	28	
**Patients**	n = 518	n = 402	
New referral requests	251	215	--
New patients seen	267	187	
Age, yr (%)			
<50	15	16	
50-64	28	29	0.889
65-79	35	37	
≥80	21	19	
Sex, female (%)	51	49	0.555
Referral eGFR, mL/min per 1.73 m^2^ (%)			
<30	18	16	
30-60	65	61	0.044
>60	17	23	

^*^ Nephrologist’s age was not collected in 2010 survey.

Nephrologists allocated a mean 8.1 (standard deviation, 5.9) hours per week to office practice with no difference between 2010 and 2012. New consults were allotted a median 60 (45–60) and follow-ups 23 (15–30) minutes. The median ratio of follow-up patients for each new patient seen was 4.08 (IQR 2.5-8.3) and 4.5 (IQR 2.2-8.3) in 2010 and 2012, respectively (p = 0.92). 27% (124/454) of patients seen in the office during the study periods experienced delays due to rescheduling. Overall, rescheduling was more often due to patients cancelling or re-scheduling (55%). Cases where the doctor re-scheduled were more common in 2010 (52%) than 2012 (34%) (p = 0.052). In 2012, 59% of BC nephrologists participated in a pooled triage system, wherein consults are assigned to the first-available nephrologist within a group of two or more. Fewer than 10% of nephrologists used central triage in 2010.

### Wait time benchmark development

Table 2 describes nephrologist and FP responses when asked to suggest maximal appropriate wait times for the 11 referral categories. When nephrologists were asked to shift between medical and patient perspectives, wait time recommendations were revised downward in 39% of responses. Condition-specific, maximally recommended wait time targets are shown in Table 3.

**Table 2:** Primary care physicians’ (FP) and nephrologists’ wait time recommendations

**Nephrology condition**	**Median wait time recommendation (weeks)**		**Proportion of nephrologists who reduced wait time recommendation ≥ 1 category when switching to patient’s perspective (%)**
	**(FP, n = 35)**	**(Nephrologist, n = 33)**	
**CKD eGFR < 30**	<3	3-6	25
**Uncontrolled hypertension**	3-6	3-6	19
**New dipstick positive proteinuria (no diabetes)**	3-6	3-6	22
**Diabetic nephropathy eGFR < 45**	3-6	6-12	42
**Diabetic nephropathy, eGFR ≥ 45**	6-12	12-24	42
**Recurrent nephrolithiasis**	6-12	12-24	36
**CKD, eGFR 30-45**	6-12	12-24	47
**New diagnosis PCKD, normal eGFR**	12-24	12-24	50
**Microalbuminuria, non-diabetic, normal eGFR**	12-24	12-24	50
**CKD, eGFR 46-60**	12-24	> 24	53
**Isolated microscopic hematuria**	Telephone	12-24	53

**Table 3:** **Recommended timeframe for nephrology assessment**^**‡**^

**Priority**	**Nephrology conditions**	**Wait time target (weeks)**
**1. Immediately threatening renal disease –** Requires rapid access	Acute kidney injury, suspected vasculitis/glomerulonephritis or Nephrotic Syndrome, eFGR < 15 ml/min	<3
**2. High risk renal disease –** Requires expedited access to prevent adverse outcomes	Diabetic nephropathy eGFR < 45 (ACr > 30 or dipstick positive)	3-6
	New onset dipstick positive proteinuria (or repeat ACRs > 30)	3-6
	CKD, eGFR < 30*	3-6
	Uncontrolled hypertension	3-6
**3. Stable renal disease –** Requires timely access	Recurrent nephrolithiasis	6-12
	Isolated microscopic hematuria	6-12
	CKD, eGFR 30-45	6-12
	New Diagnosis PCKD, normal eGFR	6-12
	Overt Diabetic nephropathy eGFR > 45	6-12
**4. At risk kidneys –** Limited empirical evidence that nephrology assessment mproves outcomes	CKD, eGFR 46-60	12-24
	Microalbuminuria, non DM, normal eGFR	12-24

‡ Telephone or other non-traditional modalities for advice may be considered as an alternative to a full office consultation at the discretion of the nephrologist.

*Median Wait Time recommended by General Practitioners fell in the < 3 wk category. In the absence of urgent features (very low GFR, rapid loss of renal function, active urine sediment, uremic symptoms, etc), such patients can safely be seen within a timeframe of 3-6 weeks.

### Waiting times

A total of 920 patients were included in the analysis. The median wait time decreased 35% from 98 (IQR 44–157) days in 2010 to 64 (IQR 21–120) days in 2012 (p = 0.0002). The median planned wait time decreased 68%, from 90 (IQR 23–139) to 29 (IQR 14–74) days (p < 0.0001). Median eGFR did not change during the waiting period in either 2010 (+ 0.59 ml/min/m2; IQR -0.44-2.45) or 2012 (+ 0.53 ml/min/m2; IQR -0.97-3.44).

**Figure 3:**
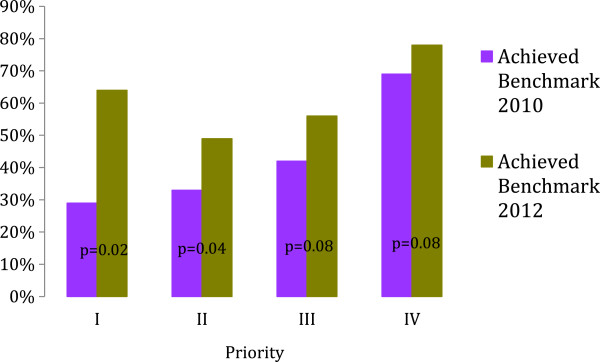
The greatest improvement in achieving recommended wait times was made for the highest priority patients (p-values are for proportion of patients in 2010 compared to 2012 waiting less than recommended benchmark within each priority category).

Figure 3 shows the proportion of new referrals that were seen within the recommended maximal wait period in 2010 as compared to 2012.

Since the hire of additional nephrologists might be expected to reduce wait times, through diluting the demand for services among more physicians, we examined wait times within health authorities that both added and did not add nephrologists. Results are shown in Table 4. There was no correlation between addition of nephrologists and reduction in wait time.

**Table 4:** Change in actual wait times within health authorities that did or did not add nephrologists between 2010-2012

**Health authority**	**Change in number of nephrologists, 2010- 2012**	**2010 Median wait (d)**	**2012 Median wait (d)**	**% Change in median wait time**
**1**	0	105	44	-58%
**2**	+1	91	95	+4%
**3**	+1	97	65	-33%
**4**	+1	267	13	-95%
**5**	+2	74	42	-43%

## Discussion

In this study, we implemented a Provincial change strategy, which invested physicians in the wait time management process. This intervention associated with a significant reduction in waiting for out-patient nephrologist consultations. Multiple factors may influence wait time dynamics, including time-varying referral rates, number of nephrologists, pooled versus individual triage systems, and practice sizes (Figure 2). We found that the proportion of patients seen within the recommended timeframes improved in the interval spanning the 2010 and 2012 data collection cycles. The fact that the magnitude of improvement aligned with referral priority (the greatest percentage reduction in wait times was for priority 1 patients), suggests that our intervention exerted an influence over nephrologists’ triaging behavior.

The 35% reduction in actual wait times (from median 98 to 64 days) was proportionately greater than the 22% reduction in total referrals (from 518 to 402, Table 1), which refutes the possibility that a reduction in referral rate fully accounts for the change in wait times. The reasons for observing a lower referral rate are unknown, but seasonal variation is not implicated because the post-implementation data collection was done over the same annual two-week period in both instances. Ongoing efforts to educate referring physicians about appropriate nephrology consultation criteria could have played a role in reducing referrals, but this effect has not been measured.

There was a net addition of five nephrologists in the Province between 2010 and 2012, which could have impacted upon wait times. However, Health authority 1 experienced a near 60% reduction in wait time, despite no net change in the number of nephrologists between 2010 to 2012, while health authority 2 had wait times increase 4% despite adding a nephrologist. Health authorities in BC are mostly closed systems with respect to patient flow, thus it is likely that nephrologist flux did not have a major impact on wait times.

With respect to triage practices, 60% of BC nephrologists had adopted the first-available nephrologist (pooled) triage model between 2010 and 2012. It is possible that efficiencies related to this practice change accounted for a share of the reduction in wait times. This is supported by the finding that planned wait times showed a marked, 68% reduction in 2012 compared to 2010. Central-triage was recommended as part of the Provincial change management strategy, which likely helped to promote this transformation.

While the evidence supports early referral for *high risk* CKD patients [9-12], our benchmarks also include recommendations for immediately threatening, stable, and at risk CKD patients (priority categories 1, 3 and 4), The need for rapid assessment is obvious for category 1 patients, but evidence supporting early referral for categories 3 and 4 is lesser [21]. Inclusion of wait time recommendations for priority 3 and 4 referrals is not intended to substitute specialist assessment for appropriate CKD screening and triage, but rather to bolster communication by establishing norms and facilitating appropriate expectations among patients and referring colleagues.

### Strengths and limitations

Our study had several noteworthy strengths. We used robust data collection methods including direct data entry at the point of care with standardized forms. The contemporary time period allowed collection of representative data including a full case mix. We also achieved a very high nephrologist participation rate. There have been few previous attempts to quantify waiting time I, and none for adult nephrology. The Fraser institute conducts annual Canadian wait time surveys, which rely upon physicians’ voluntary responses to a mailed questionnaire [22]. These data estimate median delays to medical specialist assessment in BC of 2 and 4 weeks for medical oncology and general internal medicine, respectively. Inferences based on these studies cannot be applied to nephrology specifically and are limited by lack of direct measurement as well as low response rates (17% - 28%).

With regard to benchmarks, the process used builds upon prior expert-opinion based benchmarking methodology in three ways: first, our recommendations gain added validity because patient outcomes were monitored through tracking the change in eGFR during the wait; second, we measured referring physicians’ expectations and allowed this to influence the benchmarks; third, the benchmarks quantified a surrogate for patients’ wishes by weighing in the nephrologists tendency to support shorter queues when asked to assume the patient’s perspective. In so doing, the proposed targets acknowledge the patient’s ‘cost’ associated with waiting. This cost is difficult to define, though in CKD, it may include productivity losses associated with absenteeism and presenteeism [23,24] as well as the intangible psychological impact of uncertainty when faced with a significant health concern.

Interpretation of these data must be considered in view of certain limitations. The data collected by MOAs may have been imprecise. We attempted to minimize this through manual audit by a nephrologist. In cases where the MOAs reason for referral was inconsistent with objective data, we assigned priority according to the objective data available (eGFR, amount of proteinuria etc.). Moreover, data collection personnel and methodology were largely the same for both cycles, making it unlikely that MOA imprecision would have affected the direction of overall findings.

A second limitation is that the data collection period was relatively short. This period was the maximum that could be negotiated in order to minimize disruption to MOAs workflow. The duration of data collection was also counter-balanced by a high nephrologist participation rate, which allowed a patient sample size (n = 920) large enough to draw statistical inferences.

A third limitation is that the patient perspective regarding appropriate wait time was not directly evaluated. Instead, it was weighed using the surrogate of rate at which nephrologists revised their wait time recommendations downward when assuming the patient’s perspective. The literature suggests patients are dissatisfied with waiting and their preference is to access specialists within the shortest possible timeframe [25,26]. Thus, we feel that a reduction in wait time targets through the use of a surrogate measure of the patient’s perspective is likely in keeping with the direction of patients’ true wishes.

Finally, due to nephrologist anonymity, and an inability to link nephrologists with practice groups, we were not able to conduct a sensitivity analysis examining the impact of group practice level variables, which might allow additional conclusions to be drawn from these data.

## Conclusions

An integrated, physician-led, provincial initiative to measure wait times and develop consensus waiting targets associated with improved access to outpatient nephrology consultations in BC. Improvements in the proportion of patients seen within recommended timeframes were most marked for the highest priority patients, which suggests the benchmarks had a direct influence on triaging behavior. Further research is needed to determine whether this effect is sustainable.

Future studies in this area should attempt to improve intelligent triage of CKD patients based on adverse markers such as proteinuria [27], newer and more accurate eGFR equations [28,29], or novel prediction formulas to identify progressive CKD [30]. In addition, research investigating the link between wait times for specialist assessment with short and long term outcomes are warranted.

## Competing interests

The authors declare that they have no competing interests.

## Authors’ contributions

MS, AL and MB contributed to the study design. AR and OD conducted the statistical analysis. The manuscript was prepared by MS, AL and MB. All authors reviewed and approved the manuscript.

## Pre-publication history

The pre-publication history for this paper can be accessed here:

http://www.biomedcentral.com/1471-2369/14/182/prepub
